# Do personal stories make patient decision aids more effective? A critical review of theory and evidence

**DOI:** 10.1186/1472-6947-13-S2-S9

**Published:** 2013-11-29

**Authors:** Hilary L Bekker, Anna E Winterbottom, Phyllis Butow, Amanda J Dillard, Deb Feldman-Stewart, Floyd J Fowler, Maria L Jibaja-Weiss, Victoria A Shaffer, Robert J Volk

**Affiliations:** 1 Institute of Health Sciences, University of Leeds, 101 Clarendon Road, Leeds, West Yorkshire LS2 9LJ, UK; 2 Institute of Health Sciences, University of Leeds, 101 Clarendon Road, Leeds, West Yorkshire LS2 9LJ, UK; 3 School of Psychology, University of Sydney, Brennan MacCallum Building (A18), University of Sydney, NSW 2006, Australia; 4 Department of Psychology, Grand Valley State University, 2224 Au Sable Hall, One Campus Drive, Allendale, Michigan 49401, USA; 5 Department of Oncology & Division of Cancer Care and Epidemiology, Cancer Research Institute, Queen's University, 10 Stuart Street, 2nd level, Kingston, Ontario K7L 3N6, Canada; 6 Informed Medical Decisions Foundation, 40 Court Street, Suite 300, Boston, Massachusetts, 02108, USA; 7 Office of Outreach and Health Disparities, Dan L. Duncan Cancer Center, Baylor College of Medicine, One Baylor Plaza, Suite 450A (Mail Stop BCM305), Houston, Texas 77030, USA; 8 School of Health Professions & Department of Psychological Sciences, University of Missouri, Ellis Fischel Cancer Center, DC 116.88, 115 Business Loop 70W, Columbia, MO 65203, USA; 9 Department of General Internal Medicine, Unit 1465, The University of Texas MD Anderson Cancer Center, 1515 Holcombe Blvd, Houston, Texas 77230, USA

## Abstract

**Background:**

Patient decision aids support people to make informed decisions between healthcare options. Personal stories provide illustrative examples of others’ experiences and are seen as a useful way to communicate information about health and illness. Evidence indicates that providing information within personal stories affects the judgments and values people have, and the choices they make, differentially from facts presented in non-narrative prose. It is unclear if including narrative communications within patient decision aids enhances their effectiveness to support people to make informed decisions.

**Methods:**

A survey of primary empirical research employing a systematic review method investigated the effect of patient decision aids with or without a personal story on people’s healthcare judgements and decisions. Searches were carried out between 2005-2012 of electronic databases (Medline, PsycINFO), and reference lists of identified articles, review articles, and key authors. A narrative analysis described and synthesised findings.

**Results:**

Of 734 citations identified, 11 were included describing 13 studies. All studies found participants’ judgments and/or decisions differed depending on whether or not their decision aid included a patient story. Knowledge was equally facilitated when the decision aids with and without stories had similar information content. Story-enhanced aids may help people recall information over time and/or their motivation to engage with health information. Personal stories affected both “system 1” (e.g., less counterfactual reasoning, more emotional reactions and perceptions) and “system 2” (e.g., more perceived deliberative decision making, more stable evaluations over time) decision-making strategies. Findings exploring associations with narrative communications, decision quality measures, and different levels of literacy and numeracy were mixed. The pattern of findings was similar for both experimental and real-world studies.

**Conclusions:**

There is insufficient evidence that adding personal stories to decision aids increases their effectiveness to support people’s informed decision making. More rigorous research is required to elicit evidence about the type of personal story that a) encourages people to make more reasoned decisions, b) discourages people from making choices based on another’s values, and c) motivates people equally to engage with healthcare resources.

## Background

This article examines the evidence to support the addition of personal stories to patient decision aid interventions. Patient decision aids (PtDAs) are evidence-based resources designed to help people make informed decisions between treatment, or testing, options [1]. An informed decision is one that is made well and is based upon a person’s evaluations of accurate information about the advantages and disadvantages of all the treatment options and their consequences, in accordance with the person’s beliefs and personal trade-offs between these evaluations [2]. PtDAs are complex interventions with components designed to a) provide evidence-based information presented in a way that enhances patient understanding of the health problem, treatment options and their consequences, b) structure and categorise information enabling people to attend to more details with less cognitive effort and/or bias, and c) guide people in the process of reaching a decision by making explicit their values and/or trade-offs between evaluations.

Many measures are used to assess the effectiveness of PtDAs and demonstrate they enhance patients’ informed decision making about treatment and screening options over and above usual healthcare practices [1-3]. These measures assess both a) items associated with the factors that people employ to make a decision (e.g., knowledge, perceptions of advantages and disadvantages of options, and values about health, illness, and lifestyles), and b) items associated with appraising the decision made (e.g., choice, perception of making an informed decision). In addition, PtDAs are evaluated more broadly to assess their impact on aspects of the real-world health context, such as patients’ engagement with health information and service resources, patient-professional interactions, and shared decision making [1-3].

Personal stories are narratives, testimonials, or anecdotes that provide illustrative examples of others’ experiences relevant to the decision [4,5]. Narratives provide a coherent, causal account of an experience that has occurred or that is expected to occur, giving a structure or plot to shape a person’s interpretation of an event or experience [6]. This narrative or story provides the meaning, time-line, and context of an event from the narrator’s perspective, causally linking their knowledge, beliefs, experiences, actions, and emotions with social and ethical mores of relevance to that person [7-9]. Personal stories, narratives, and anecdotes are an everyday medium that people use to communicate information to others [8,9]. Within health, illness scripts and narratives are deemed to be a valued resource to enable understanding of a patient’s experience of an illness and the impact of an illness on the patient’s life and wellbeing [4,5,8-10]. Narratives are perceived as providing essential emotional and social information not usually found within routine resources that lend meaning and perspective to a patient’s predicament [7]. Evidence indicates another patient’s experience is important to a significant proportion of patients, helping them understand their illness, cope and adjust to treatment regimens, and navigate various healthcare systems [11-13]. However, there is limited evidence on how useful patients find another patient’s story when making a decision between healthcare options [5,10].

For those developing interventions to inform patients about health promotion initiatives intended to meet public health objectives, narrative communications are seen as a promising way of encouraging people to change their health behaviours [14]. Additionally, communicating information within personal stories is seen as particularly beneficial to subgroups of people who may have lower levels of literacy and numeracy [7,13,15-20], and/or come from cultures with strong oral traditions [14], as stories may help people’s learning by providing a model from which attitudes and skills can be emulated [15]. However, findings indicate that narrative communications affect differentially the measures of people’s decision-making processes and decision outcomes, compared with non-narrative prose and/or statistical information [4,5,11,21-37]. It is equally plausible that these findings are evidence of people making their decisions based on another’s values or choices as it is that people are being supported to make informed decisions between options. What is unclear is why and how the addition of others’ personal stories to PtDAs interventions affects their ability to support patients in making more informed decisions than would result from exposure to PtDAs without personal stories or standard healthcare information.

Within healthcare, “others” can include patients, carers, family, and professionals. Personal stories may be told from the first-person (e.g., the patients’ or carers’ accounts of their own experience) and the third-person (e.g., the professionals’ or carers’ account of the other’s experience) perspectives, and in the form of representations of discussions between the patient and another person (e.g., patient plus professionals and/or carer); although stories told in the first-person tend to be twice as persuasive as those in the third-person [4]. Stories can be presented as text in leaflets (with and without photos and/or images), in audio and video clips (via DVDs and/or web resources), and in face-to-face encounters (individually or in groups). Personal stories can be delivered by the person whose story it is or by an actor.

The following are types of personal stories that could be included within patient resources:

• Testimonials of patients (or actors) talking about their experiences relevant to the decision.

• Scripted narratives of patients (or actors) talking about their experiences relevant to the decision.

• Narratives by health professionals (or actors) describing their patients’ decision making experiences.

• Documentaries (real or enacted) narrating the experience of patients with professionals and others (e.g., family, carers) when making decisions about healthcare options, usually with photographs or video stills.

• Conversations (real or enacted) illustrating the interaction between patients with other parties (e.g., health professionals, family, or carers) when making decisions about healthcare options.

## Theoretical justification

Decision science, health psychology, and communication theories provide guidance on the cognitive, emotional, and motivational factors associated with people’s treatment decision making and their health and illness behaviours [11,15,18,22,38,39]. Explanations of how people process information to make decisions generally agree that there are two inter-related systems used: an experiential-automatic process (“system 1”) that is quick, effortless, and does not require deliberation before action (e.g., relying on an heuristic or rule of thumb such as trust in another’s judgments); and an analytic-deliberative process (“system 2”) that is effortful, cognitively demanding, and requires active reasoning before action (e.g., weighing up the advantages and disadvantages of all options).

Studies exploring the effect of communicating information in different ways on peoples’ risk perceptions and intended behaviour indicate that: a) people make choices based on integrating both factual prose and narrative information; b) narrative information influences people’s choice directly (system 1) and in-directly via cognitions such as judgements about risk and values (system 2); and c) the persuasive effect of narrative, or statistical, information varies with reference to the initial perspective of the decision maker [40,41]. In other words, people’s individual characteristics and experiences will affect what information they focus on when representing the decision and how they reason about it when it is packaged in different ways.

It is system 2 strategies that are associated with awareness of making a decision well, searching for information about all options, actively engaging with the process of reaching a decision based on the option details, reasoning about the advantages and disadvantages to them of the options (i.e., counter-arguing), and making a choice based on one’s own evaluations rather than someone else’s judgment or recommendation. As mentioned, the function of PtDAs is to encourage active thinking about the decision options and to support patients in making informed decisions—that is, to encourage use of system 2 strategies. So, patients employing mainly system 1 strategies are less likely to make an informed decision between healthcare options. Evidence indicates that aspects of the type, structure, and content of personal stories encourage people to employ automatic strategies (system 1) when processing the decision information—for example: the credibility of the narrator [25]; the affective and/or value terms used within the story [26-29]; the social references a narrator provides implicitly when telling a story [15,30,31]; and the temporal and causal framework provided implicitly by having to link together events in order to tell a story [32-34]. Equally, particular aspects of narrative communications have been identified as encouraging deliberative processing (system 2)—for example: increasing motivation to attend to the information [35]; making the information more salient and memorable [30,31]; and modelling the process of decision making through depiction of interactions with others [20]. Consequently, it seems that information communicated within personal stories may be as likely to hinder patients’ informed decision making as to facilitate it (see Table 1).

**Table 1:** Examples of the Biasing or Facilitating Influence of Facts within Personal Stories.

Decision Aid Function	Biasing by Personal Stories	Facilitating by Personal Stories
Accurate and Balanced Information	Narrator uses more (or less) value-laden and emotional terms to describe (less) favoured options.	Narrator uses language that helps describe the emotional content of the options and decision.
Accurate and Balanced Information	Narrator refers to only those facts important to him/her in choosing option A or rejecting option B.	Narrator makes explicit the importance of exploring all options regardless of prior experiences.
Attention and guidance	The smaller selection of facts used by the narrator to explain his/her choice is easier to process and evaluate.	Guidance on how the narrator went about making the decision.
Attention and guidance	The story primes/ reinforces selected facts interfering with processing of all facts.	Presents facts in a more accessible way, making them easier to process and recall.
Patient evaluations/trade-offs	Processing the values and trade-offs important to the narrator when s/he made their choice.	Helps make explicit the role of different patients’ values and experiences to make the decision.
Patient evaluations/trade-offs	Patient opinions about the narrator, and not the story content, used to make the choice.	Provides relevant social reference and/or causal information to help patients reach judgments.

Developers of interventions for health education and health behaviour change argue that information packaged within personal stories is more persuasive than non-narrative communications and such stories are, therefore, a useful way of presenting information to patients [14]. Some of the mechanisms explaining the disproportionately persuasiveness of narrative communications are:

a) *memory* – supplementary narratives within health information interrupt people’s processing, and recall, of the factual information [41];

b) *evaluation* – narrative information is less threatening than statistical information and so circumvents the initiation of defensive responses associated with negative affect [40], or narrative information is integrated by automatic processes and so reduces counter-arguing and is more difficult to discount analytically, or narrative information is given more weight because of the narrative’s characteristics (e.g., emotional content) [40,41]; and

c) *dual-process controller* – if narrative and statistical information are processed differently, then there is likely to be an interplay between the judgments made from evaluating statistical or narrative information first, and the person’s motivation to attend to and assimilate further information [41].

What developers of standardised information need to consider when introducing information packaged as a personal story is that, because of patients’ personal experiences and values, the decision maker will respond unpredictably to the narrator of the story. In other words, the standardised information is including an element that is likely to cause variation in the information that the decision maker will attend to, process, represent, and act upon based on differences in his/her values and experiences.

## Aim

Given this complex background and range of theoretical arguments, our review aims to synthesise the evidence of empirical studies published since the Butow et al. 2005 review [5] evaluating PtDAs, in order to ascertain if the addition of a personal story component enhances informed decision making.

## Methods

### Design

We carried out a survey of empirical studies comparing the effect of a PtDA intervention with and without a personal story component on people’s healthcare decision making, using a systematic review method.

### Search strategy

We searched for all articles with a decision making term (decision making / decision support / decision aid / decision theory, choice, preference) and a personal story term (narratives, patient stories, anecdotes, testimonials, exemplars).

### Searches

Searches were carried out in two electronic databases, Medline and PsycINFO (2005-2012), identifying 734 articles. Additionally, the reference lists of relevant reviews were searched [1,4,5,10,11,42,43], key authors contacted, and/or an author search was carried out using Google Scholar. All authors contributed to the identification of key papers, authors, and search terms. AEW carried out the searches. All authors contributed to the inclusion and exclusion criteria of articles within the review. AEW and HLB discussed all decisions of included articles, 5% of the excluded article decisions, and all borderline decision articles until consensus was reached.

### Inclusion criteria

We searched for studies: a) evaluating the effects of a personal story component of a PtDA intervention on people’s healthcare decision making, b) involving individuals making real or hypothetical decisions; c) presenting personal stories in the first or third person; d) using experimental and/or RCT designs, before-and-after study designs, and/or cohort study designs; e) involving an adult population; and f) published in English.

### Exclusion criteria

We excluded discussion and/or review papers, as well as studies a) assessing message-framing only; b) using video or verbal (narrated) formats for information delivery only; c) involving proxy decision making (i.e., decision making by an individual for another); and d) using non-experimental or single-case study designs.

### Data extraction

The following data were elicited systematically from each article: paper characteristics; design, sample, method and measures; theoretical framework; study aim; story content, integration within the PtDA and characteristics of narrative; and study findings.

AEW and HLB extracted the information for all articles; the other authors checked the accuracy of the data elicited.

### Analysis

The findings are described and integrated using a narrative synthesis.

## Results

Of 734 citations, we identified 11 articles reporting findings from 13 studies investigating the effect of a PtDA with or without a personal story on people’s healthcare decisions (see Additional file 1 - Table S1).

### Contexts

The health contexts were: screening for prostate [18], breast [19], and colorectal cancer [28]; treatment decisions between angioplasty / bypass surgery [44], mastectomy / breast conserving surgery [45,46], peritoneal dialysis / haemodialysis [47]; and end-of-life level of care in cancer [47] and in dementia [48-50]. PtDA interventions were delivered directly via access to web links [19,28,44,45,47] or face-to-face with computer support [18,46,48-51]. Most studies assessed hypothetical choices or preferences [28,44,45,47-51] (see Additional file 1 - Table S1).

### Story types

The personal story types were: first-person scripted narrative communications [44,45,47] tailored to the characteristics of the decision makers [19,28]; third-person scripted narrative describing other patients’ experiences [47]; documentary [48-51] illustrating the illness state and types of care; and conversations illustrating the interaction between patients and others [18,46] (see Additional file 2 - Table S2).

The personal stories varied in their content, delivery, and length. All personal stories provided information about the narrator’s perception of making the decision and the health context that reinforced aspects of the PtDA information of importance to the narrator. Some stories included additional information about the treatment options, disease severity, and associated consequences from the options presented in the PtDA information [44-47]. Some included additional information on engaging with health professionals and services, and interactive exercises from the PtDA [18,51].

### Measures

See Additional file 1 - Table S1. Measures exploring the effect of personal story communications within PtDAs included items about:

a) Decision outcome (e.g., preference, choice and/or uptake of treatment / test);

b) Participants’ perception of a decision made effectively (e.g., decisional conflict, satisfaction, certainty and/or regret);

c) Factors participants used during decision making (e.g., judgments and values of the decision options and consequences, perceptions of the health problem, reasoning, knowledge);

d) Individual differences in participants’ abilities and/or experiences (e.g., literacy, numeracy, decision-making style, self-advocacy);

e) Engagement with others (e.g., friends and family, health professionals and services); and

f) Acceptability of the PtDA resource (e.g., trustworthy, credible, useful, likeable).

### Study objectives

All studies compared PtDAs designed to inform people about health and illness and to support their experiences of healthcare (see Additional file 1 - Table S1). The studies had three broadly different aims and frameworks guiding their research. First, some studies were primarily concerned with investigating whether or not narrative communications bias and/or facilitate informed decision making between treatment options. Second, some studies were primarily concerned with investigating whether or not narrative communications can encourage people to have a test or treatment. Third, some studies were primarily concerned with whether or not narrative communications enable informed engagement with, or education about, health services and treatment options. The studies’ results are synthesised in Table S3 (Additional file 3). Below, we describe these three kinds of studies in more detail.

### The informed decision making studies

Fagerlin et al. (2005) investigated the single and combined impact of patient anecdotes (written) and statistical information within a written PtDA about angina and treatment options [44]. Winterbottom et al. (2011) investigated the single and combined impact of a patient and professional’s story (video / written), and decision-attribute information summary table, within a written PtDA on haemodialysis and peritoneal dialysis options [47]. Shaffer et al. (in press) investigated the impact of a patient story, and individual differences in e-health literacy, within a video PtDA on lumpectomy and mastectomy options [45].

For the written PtDAs, the inclusion of narrative information was more likely to encourage participants to make a choice based on the narrator’s choice. Pictograms helped counter this effect for statistical information [44], but the table summary of information did not counter this effect for prose [47]. Both written and video PtDAs observed differences in participants’ reasoning about, and judgments of, the risks and benefits of options when they received the personal story enhanced resources [44,45,47]. For participants receiving only the personal story-enhanced video, there was a differential effect—by individual differences in e-literacy ability—in perceived emotionality and trustworthiness of the PtDA [45].

### The informed choice studies

Kreuter et al. (2010) investigated the effect—on mammography uptake, cancer beliefs, and PtDA evaluation—of a video PtDA made by organizing clips of personal stories from 36 women into a collective narrative about breast cancer and mammography (“living proof”), compared with a video PtDA presenting the same factual content as in the living proof video, but narrated by an actress [19]. Dillard et al. (2010) investigated the effects—on people’s knowledge, perceived risks, values, and interest in having colorectal cancer screening—of a written PtDA with or without a personal story with photographs matched to participant characteristics [28].

All studies found participants viewing personal stories had increased cancer fears, decreased perceived barriers to screening, and increased interest in screening compared to those viewing the non-narrative decision aid. There were no differences in knowledge scores after viewing the PtDAs but, over time, there was a greater recall of facts within the personal-story-enhanced resource.

### The informed engagement studies

Volk et al. (2008) investigated the effects—on the acceptability of the PtDAs, knowledge, decisional conflict, and self-advocacy—of an audio-booklet PtDA for prostate cancer screening compared with an entertainment-education (“edutainment”) [52] PtDA containing interactive learning modules, animation, and soap-opera telenovela stories [18]. Jibaja-Weiss et al. (2010) investigated the effects on choice, knowledge, decisional satisfaction, and conflict of a written breast cancer and treatment options PtDA with an edutainment resource [46]. Volandes (2009, 2009b, 2011) investigated the effects on participants’ treatment choice of a narrated script (delivered by the researcher) about end-stage dementia and three levels of health care treatment (life-prolonging; limited; comfort) with or without a six-minute video that included a) images and narratives about an elderly women with end-stage dementia and her family in a care home and b) elements of the three different levels of care [48-50]. El-Jawahri (2011) investigated the effects—on participants’ care and resuscitation choices, decision uncertainty, and knowledge—of narrated script (delivered by the researcher) about advanced cancer and three levels of care (life-prolonging; limited; comfort) with or without a six-minute video including images and narratives of patients experiencing elements of the three different levels of care [51].

For all of these studies, participants viewing the personal-stories-enhanced PtDAs made different decisions from those viewing the non-narrative PtDAs [46,48-51]. The pattern of findings differed between the edutainment-enhanced and video-enhanced studies. For the edutainment studies, all the PtDA groups increased knowledge and decreased decisional conflict [18,46]; prior to surgery, women receiving the breast cancer treatment edutainment PtDA were clearer about their personal values and felt more informed [46]. For the video studies, participants viewing the narrative-enhanced PtDAs had higher knowledge, but higher decisional uncertainty [51], than those receiving the narrated script [48,51]. There was some evidence that narrative-enhanced PtDAs were differentially effective with those of differing health literacy levels [18,48].

### In summary

Participants made different judgments and decisions about the same evidence-based information within the PtDA when they were exposed to a personal story of another patient’s experience of decision making and/or health problem. When the non-personal-story PtDA and the PtDA with a personal story appeared to have equivalent informational content, participants’ knowledge scores were the same—that is, they were equally facilitated [19,28,46]. There is some suggestion that a personal story component of a PtDA may help participants’ recall information and support discussing the decision with others at a later time [18,19,46].

The findings suggest that personal stories within PtDAs are affecting both system 1 and system 2 strategies, and do so differentially by individual differences in literacy and numeracy. The following review findings are suggestive of system 1 processing: less counterfactual reasoning; changes in emotional reaction to the PtDA (e.g., it’s likable, comfortable); and greater ratings for emotion-laden perceptions such as fear of disease or appearance. The following review findings are suggestive of system 2 processing: greater ratings about making decisions deliberatively and/or informed and/or based on own values; decisions based on trade-offs between perceived advantage and disadvantages of all options; stability of decisions, knowledge, and values over time; and greater ratings of the PtDA helping to make decisions deliberatively (e.g., it’s useful). There was insufficient evidence reported within studies to ascertain if the addition of personal story information was more likely to encourage people to make decisions based on another’s judgments or choice (system 1) or more deliberative reasoning (system 2) than the PtDA alone. Currently, this pattern of findings was observed in studies investigating both experimental-hypothetical and real-world decisions.

## Discussion

There is a paucity of research investigating systematically the impact of a PtDA with or without a personal story on people’s healthcare decision making. Within this review, there was variability in the way personal stories were sourced, constructed, delivered, and integrated within the intervention. Additionally, the study purpose, and therefore the function of the personal story, varied in terms of investigating biases in and facilitators of decision making, health behaviour change, or participant engagement with information. The studies employed measures consistent with the studies’ aims, but not sufficient to ascertain if the personal-story-enhanced PtDA was more able to help people make more informed healthcare decisions than the PtDA intervention alone. As a result, the synthesis was not able to identify the active ingredients of the personal story that may facilitate, or limit, the effectiveness of PtDA interventions to support people making informed decisions between treatment options. However, there were some similarities across the experimental and real-world studies, suggesting people use both personal story and factual information when making healthcare decisions, and that there is an interplay between the processing of personal stories and factual information with subsequent attention to information, evaluations, and decisions [35,37-40,45,47]. It was unclear how this interplay could be maximised to ensure patients’ healthcare decision making is supported rather than biased.

Within the review, some studies assessed individual differences in health literacy [18,19,45,46,48-51] to investigate whether the inclusion of personal stories within PtDAs may redress differences in people’s health literacy and, ultimately, lead to greater equity in healthcare utilisation. Health literacy is a broad, multifaceted concept describing the cognitive and social skills and/or abilities determining a person’s motivation to access, understand, and use information to promote and maintain health [see 43]. The studies exploring these individual differences by intervention effects have mixed and moderate results, in part because a) few studies compared the same PtDA intervention delivered in the same way with just the addition, or omission, of the personal stories component [19,46], and b) authors used different measures to assess different aspects of health literacy.

However, three patterns emerged. First, well-designed PtDAs contain the active ingredients associated with good health literacy interventions without the need to include personal stories [43]. Second, adding personal stories interacts with individual differences in literacy such that people with lower literacy levels respond more positively towards the use of narratives in PtDAs, whereas those with higher literacy respond more negatively to their inclusion [18,45,46]. Third, the addition of personal stories seems to affect the motivational and/or emotional elements associated with attending to, and recall of, information [19,46]. It may be that the content and structure of PtDAs are already sufficient to address inequities in health numeracy and literacy skills, but personal stories enhance motivation to engage with, and perceived experience of using, the resources in some populations.

There is tremendous scope to advance understanding of the role of personal stories in patient decision making and in healthcare behaviour change. It is possible that patient stories enable PtDA developers to design evidence-based resources that increase healthcare engagement and support patients in making informed decisions. However, we need to recognise that personal stories are complex interventions with component parts that affect people’s judgements and actions independently of factual information. To understand what are the active components within stories used in healthcare information, and whether they facilitate or bias decision making, authors need to describe in more detail their structure, content, and function. Shaffer and Zikmund-Fisher [16] are developing a taxonomy to guide those developing and/or using personal stories within healthcare information to think critically about its purpose (e.g., to inform, engage, model behaviour, persuade, or comfort) and content (e.g., the process of decision making; the decision experience; the decision outcome). In addition, authors should consider explicitly the mechanism or rationale explaining why the story impacts on a patient’s awareness, understanding, and/or assimilation of the decision information differentially from non-narrative or factual information.

## Conclusion

This review of studies indicates that people engage differently with a PtDA when it includes a personal story component. PtDA interventions are evidence-based resources designed to enable patients to reason about the relevant evidence-based information to make an informed decision. Personal stories contain complex and detailed information about, usually, one (or more) person’s experience with an illness, with a decision-making process, and with the consequences of making a healthcare choice. The narrative both structures and interprets the information so that it makes sense from the narrator’s perspective. Packaging information as a story affects people’s use of both system 1 (intuitive-experiential) and system 2 (deliberative-analytic) information-processing strategies. Research studies need to be designed so as a) to assess the effect of a patient story on people’s decision making over and above that of a PtDA, using measures to gauge the effectiveness of a PtDA in supporting people’s informed decision making, and b) to understand the effects of different ways of communicating information on the people’s decision-making strategies. It is essential to the field to elicit evidence illustrating what type of personal stories are a) more likely to motivate people to engage with PtDAs to make reasoned decisions (system 2), and b) less likely to make a choice based on another’s values (system 1). Measures currently used to evaluate improvements in decision quality and outcome when comparing PtDAs and usual care practices [3] may not be sufficient to elicit evidence of the impact of different components within PtDA interventions on the system 1 and system 2 strategies that people employ when making decisions. Without this evidence, it is unclear if the addition of a personal story enhances or counters the effectiveness of a PtDA to support patients’ informed decision making.

## List of abbreviations used

PtDA: patient decision aid; RCT: randomized controlled trial

## Competing interests

Deb Feldman-Stewart has received travel support to teach a course on designing evidence-based decision aids from the Informed Medical Decisions Foundation, a not-for-profit (501 (c)3) private foundation (http://www.informedmedicaldecisions.org). The Foundation develops content for patient education programs. The Foundation has an arrangement with a for-profit company, Health Dialog, to co-produce these programs. The programs are used as part of the decision support and disease management services Health Dialog provides to consumers through health care organizations and employers. Victoria A. Shaffer and Robert J. Volk have also received research funding, travel support and honoraria from the Informed Medical Decisions Foundation.

All other authors declare that they have no competing interests.

## Authors’ contributions

All authors (HLB, AEW, PLB, FJF, RJV, VAS, MLJ-W, DF-S, AJD) made substantial contributions to the conception and design of the scoping review, were involved in drafting the manuscript, providing critical review and adding important intellectual content, and approved the final version for publication. In addition, AEW was responsible for carrying out the search of the empirical papers, extraction of data, and assimilation of evidence within tables; and HLB was responsible for co-ordinating the team, drafting the manuscripts, and integrating reviewers’ comments.

## Supplementary Material

Additional file 1 **Table S1:** Studies Included in the Review: Methodological Detail Click here for file

Additional file 2 **Table S2:** Characteristics of Personal Stories used in Reviewed Studies Click here for file

Additional file 3 **Table S3:** Summary of Reviewed Studies’ Findings on Decision Processes and Outcomes Click here for file

## Declarations

Publication of this supplement was partially funded by an unrestricted grant from the Informed Medical Decisions Foundation. The remaining cost was funded by Leeds Institute of Health Sciences, School of Medicine, University of Leeds (Bekker). Administrative and editorial support for this supplement was also provided by The University of Texas MD Anderson Cancer Center.

This article has been published as part of *BMC Medical Informatics and Decision Making* Volume 13 Supplement 2, 2013: The International Patient Decision Aid Standards (IPDAS) Collaboration’s Quality Dimensions: Theoretical Rationales, Current Evidence, and Emerging Issues. The full contents of the supplement are available online at http://www.biomedcentral.com/bmcmedinformdecismak/supplements/13/S2.
