# Supplementary material for: Do personal stories make patient decision aids more effective? A critical review of theory and evidence
Source: BMC Med Inform Decis Mak. 2013 Nov 29;13(Suppl 2):S9. doi: 10.1186/1472-6947-13-S2-S9 (PMC4044102; doi:10.1186/1472-6947-13-S2-S9)
Supplement: Additional file 1 — Table S1: Studies Included in the Review: Methodological Detail [file 1472-6947-13-S2-S9-S1.pdf]

Table S1. Studies Included in the Review: Methodological Detail

Authors, Year, Citation Number	Sample	Theory	Aim	Measures
Dillard et al. (2010), [1]	N = 1533 General population, USA	Affective forecasting theory	To assess the use of patient stories in promoting colorectal cancer screening	Perceived barriers of screening; risk perception; knowledge; screening interest; time spent reading text.
Volandes et al. (2009), [2]	N = 200 General population, USA	None stated	To assess the use of a video of a patient with advanced dementia on end of life care choices.	Hypothetical end of life care; knowledge and experience of dementia; health literacy; acceptability video.
Volandes et al. (2009b), [3]	14 pairs of patients and their carer. General population, USA	None stated	To compare concordance of preferences among elderly patients and their carers.	Hypothetical end of life care; knowledge and experience of dementia; health literacy; acceptability video.
Volandes et al. (2011), [4]	N = 76 General population, USA	None stated	To assess the use of a video of a patient with advanced dementia on end of life care choices.	Hypothetical end of life care; knowledge and experience of dementia; health literacy; acceptability video.
Winterbottom et al. (2011), [5]	N = 1694 Students, UK	Information processing theory	To assess whether another patient's dialysis story affects people's choices and/or the moderating effect of a decision-attribute table.	Hypothetical decision. Knowledge and experience with kidney disease.
El-Jawahri et al. (2009), [6]	N = 50 Patients with	None stated	To assess a video of images of patients on three levels of	Hypothetical end of life care and CPR; knowledge; certainty with

	malignant glioma (cancer), USA		medical care (life-prolonging, basic, and comfort care) for advanced cancer on comfort care choices.	decision; acceptability video.
Kreuter et al. (2010), [7]	N = 489 African-American women from general population, USA	Information processing and behaviour change theories	To assess the effects of narrative and informational videos on mammography uptake.	Mammography decision; knowledge and experience breast cancer; medical mistrust; perceived physician recommendation; beliefs about cancer; perceived barriers mammography; perceived risk; acquisition information; reasoning (counter-arguing); talking to family and friends.
Jibaja-Weiss et al. (2011), [8]	N = 100 Female patients with early stage breast cancer, USA	Communication theories under the rubric of entertainment-education	To evaluate an entertainment-based decision aid for early stage breast cancer surgery in low health literacy patients.	Treatment preference; knowledge of breast cancer and treatments; decisional, conflict, satisfaction.
Volk et al. (2010), [9]	N = 450 (n = 149, low literacy; n = 301, high literacy) Patients eligible for prostate cancer screening, USA	Communication theories under the rubric of entertainment-education	To evaluate an entertainment-based decision aid for prostate cancer screening amongst high and low health literacy patients.	Acceptability and engagement with the PtDA; knowledge of prostate cancer and screening; decisional conflict scale; self-advocacy scale.
Fagerlin et al. (2005), [10]	1st study, N = 613 2nd study,	Information processing theory	To assess the effect of graphical representations of statistics reduces the influence	Hypothetical treatment decision.

	N = 875<br>General population, USA		of narrative information on decision making about treatment for angina (surgery/ balloon angioplasty).	
Shaffer et al.<br>(in press 2013), [11]	N = 200<br>General Population, USA	Information processing theory	To assess the influence of patient stories on decision making about surgery for early-stage breast cancer and resource utility.	Hypothetical decision; decisional confidence/ certainty; electronic health literacy, numeracy, decision making style; PtDA utility, trustworthiness.

References

1. Dillard AJ, Fagerlin A, Zigmund-Fisher BJ, Ubel PA: **Narratives that address affective forecasting errors reduce perceived barriers to colorectal cancer screening.** *Soc Sci Med* 2010; 71: 45-52.
2. Volandes AE, Paasche-Orlow MK, Barry MJ, Gillick MR, Minaker KL, Chang Y, Cook EF, Abbo ED, El-Jawahri A, Mitchell SL: **Video decision support tool for advance care planning in dementia: randomised controlled trial.** *BMJ* 2009; 338: b2159.
3. Volandes AE, Mitchell SL, Gillick MR; Chang Y, Paasche-Orlow MK: **Using video images to improve the accuracy of surrogate decision making: a randomized controlled trial.** *J Am Med Dir Assoc* 2009b; 10; 575-580.
4. Volandes AE, Ferguson LA, Davis AD, Hull NC, Green MJ, Chang Y, Deep K, Paasche-Orlow MK: **Assessing End-of-Life Preferences for Advanced Dementia in Rural Patients Using an Educational Video: A Randomized Controlled Trial.** *J Palliat Med* February 2011, 14(2): 169-177.
5. Winterbottom AE, Bekker HL, Conner M, Mooney A: **Patient stories about their dialysis experience biases others' choices regardless of doctor's advice: an experimental study.** *Nephrol Dial Transplant* 2011; 27(1): 325-331.
6. El-Jawahri A, Podgurski LM, Eichler AF, Plotkin SR, Temel JS, Mitchell SL, Chang Y, Barry MJ, Volandes AE: **Use of video to facilitate end-of-life discussions with patients with cancer: a randomized controlled trial.** *J Clin Oncol*. 2010; 28: 305-311.
7. Kreuter MW, Holmes K, Alcaraz K: **Comparing narrative and informational videos to increase mammography in low-income African American women**. *Patient Educ Counsel* 2010; 81 S1: S6-S14 (2010)

8. Jibaja-Weiss ML, Volk RJ, Granchi TS: **Entertainment education for breast cancer surgery decisions: A randomized trial among patients with low health literacy** *Patient Educ Counsel* 2011; 84: 41-8.

9. Volk RJ, Jibaba-Weiss ML, Hawley ST, Kneuper S, Spann SJ, Miles BJ, Hyman DJ: **Entertainment education for prostate cancer screening: a randomized trial among primary care patients with low health literacy.** *Patient Educ Counsel* 2008; 73:482-489.

10. Fagerlin A, Wang C, Ubel PA: **Reducing the influence of anaecdotal reasoning on people's health care decisions: is a picture worth a thousand statistics?** *Med Decis Making*. 2005; 25: 398-405.

11. Shaffer VA, Tomek S, Hulsey L: The effect of narrative Information in a publicly available patient decision aid for early-stage breast cancer. *Health Commun* 2013 Feb 5 [Epub ahead of print].
