# Supplementary material for: Do personal stories make patient decision aids more effective? A critical review of theory and evidence
Source: BMC Med Inform Decis Mak. 2013 Nov 29;13(Suppl 2):S9. doi: 10.1186/1472-6947-13-S2-S9 (PMC4044102; doi:10.1186/1472-6947-13-S2-S9)
Supplement: Additional file 2 — Table S2: Characteristics of Personal Stories used in Reviewed Studies [file 1472-6947-13-S2-S9-S2.pdf]

Table S2. Characteristics of Personal Stories used in Reviewed Studies

Authors, Year, Citation Number	Story Content	Integration of Story in PtDA	Other Features	Experimental Groups
Dillard et al. (2010) [1]	Patient story described one individual's experience of deciding about screening; no additional factual information	Patient story integrated into standard information in 4 sections focusing on: feeling uncertain about the decision; barriers to screening; impact bias.	Photograph of narrator matched to participants' sex, age and race.<br>Information obtained from National Cancer Institute online booklet	2x2 design:<br>Patient story (present or absent)<br>versus<br>Screening type (colonoscopy or virtual colonoscopy).
Volandes et al. (2009a, b; 2011) [2-4]	Dementia patient shown as unable to communicate, in a wheelchair, unable to feed herself.	Video delivery of decision support tool presented on a computer (lasting 2 minutes).	Picture stills of a real patient with advanced dementia (80 year old female) and 2 daughters in a nursing home setting.<br>Video developed in consultation with specialists in dementia and Functional Assessment Staging (FAST) 7a (i.e., criteria for the most advanced stage of dementia).	Verbal information (consultation)<br>versus<br>Decision support video + verbal information (consultation).
Winterbottom et al. (2011) [5]	Four scripted scenarios. Two patient and two doctor stories describing haemodialysis (HD) and continuous cycling peritoneal	Written or video delivery delivered after standard factual information about HD and CCPD.<br>In study 2, only video delivery of story plus	In study 1: 1 male and 1 female actor played 2 roles each.<br>In study 2: 2 male and 2 female actors played each of the 4 roles.<br>Narratives developed from the	Study 1: 2x2x2 format (written vs. video) x source (patient vs. doctor) x order (HD information vs. PD information).<br>Study 2: 3x2x2x2: decision table (none, before or after

	dialysis (CCPD), decision making process, and dialysis outcome (fitting into a patient's life).	additional summary decision-attribute table before or after story.	literature, study describing patient dialysis decision making, and renal specialists.	story) x order (patient vs. doctor first) x sex of patient (male, female) x sex of doctor (male, female). Control group read only basic factual information in both studies.
El-Jawahri et al. (2009) [6]	Three levels of care described, identical to the verbal information (consultation) but with visual images of patients in different scenarios.	A 6-minute video presented on a laptop computer.	Verbal narrative describing 3 levels of medical care for advanced cancer. Pictures used of patients in intensive care being ventilated by a professional.<br>Video developed in consultation with cancer specialists, filmed without prompts.	Verbal information (consultation)<br>versus<br>Verbal information (consultation) plus video.
Kreuter et al. (2010) [7]	Both videos addressed 11 key messages about breast cancer.<br>Video 1: personal stories from African American breast cancer survivors.<br>Video 2: didactic, expository form from an African American woman, cancer survivor.	Videos presented via a mobile communication research facility in the community.	Video 1: 57 short stories elicited from 29 cancer survivors. Edited to form narrative about risk, talking about cancer and mammography screening.<br>Video 2: Material developed from video 1 and presented by a single narrator with images, photos, graphics and music.<br>Videos piloted for equivalence.	Narrative video<br>versus<br>Factual prose video.
Jibaja-Weiss et al. (2011) [8]	Factual information about surgery options	Decision-making process for making a	In the PtDA the storyline does not reveal what surgery option	Intervention: a computerised interactive multimedia PtDA

	and other treatments presented in interactive learning modules component of ptDA	surgery decision presented in the format of didactic soap operas	the main character chooses; a values clarification exercise is included.<br>PtDA conforms to IPDAS criteria.	utilising entertainment (soap opera episodes and interactive learning modules).<br>Control: only usual care.
Volk et al. (2008) [9]	Factual information about prostate cancer and screening presented in the format of didactic soap operas and interactive learning modules.	A computer-based PtDA based on the Edutainment Decision Aid Model (EDAM).	In the PtDA, ethnicity of main character tailored to the viewer; celebrity testimonials and values clarification exercise included.	Intervention: an interactive computer-based PtDA.<br>Control: receive an audio booklet with no values clarification, entertainment component, testimonials, or values-clarification exercise.
Fagerlin et al. (2005) [10]	The experience of former patients with each of the treatment options was presented with the scenario.	Written patient narratives with the number of narratives varied to be representative or non-representative of cure rate.	Information in the narratives was repetition of that presented in the scenario so no new information was supplied in the narratives.<br>Study 1: A pictograph and trade-off quiz were also presented to some participants.	Study 1: 2 (representative vs. non-representative narratives) x 2 (pictograph and quiz vs.. no pictograph or quiz).<br>Study 2: 2 (pictograph vs. no pictograph) x 2 (no trade off quiz vs.. trade off quiz) [all participants received non-representative narratives]
Shaffer et al. (in press 2013) [11]	Three topics including patients' reaction to diagnosis; strategies for making the decision; the least and most important aspects of treatment for the patient.	Video clips to supplement factual information talked about by the narrator.	Verbal stories from 12 patients' experiences of treatment for breast cancer. All women were middle-aged, 9 were Caucasian and 3 African-American	Video resource<br>versus<br>Video resource plus personal stories

References

1. Dillard AJ, Fagerlin A, Zigmund-Fisher BJ, Ubel PA: **Narratives that address affective forecasting errors reduce perceived barriers to colorectal cancer screening.** *Soc Sci Med* 2010; 71: 45-52.
2. Volandes AE, Paasche-Orlow MK, Barry MJ, Gillick MR, Minaker KL, Chang Y, Cook EF, Abbo ED, El-Jawahri A, Mitchell SL: **Video decision support tool for advance care planning in dementia: randomised controlled trial.** *BMJ* 2009; 338: b2159.
3. Volandes AE, Mitchell SL, Gillick MR; Chang Y, Paasche-Orlow MK: **Using video images to improve the accuracy of surrogate decision making: a randomized controlled trial.** *J Am Med Dir Assoc* 2009b; 10; 575-580.
4. Volandes AE, Ferguson LA, Davis AD, Hull NC, Green MJ, Chang Y, Deep K, Paasche-Orlow MK: **Assessing End-of-Life Preferences for Advanced Dementia in Rural Patients Using an Educational Video: A Randomized Controlled Trial.** *J Palliat Med* February 2011, 14(2): 169-177.
5. Winterbottom AE, Bekker HL, Conner M, Mooney A: **Patient stories about their dialysis experience biases others' choices regardless of doctor's advice: an experimental study.** *Nephrol Dial Transplant* 2011; 27(1): 325-331.
6. El-Jawahri A, Podgurski LM, Eichler AF, Plotkin SR, Temel JS, Mitchell SL, Chang Y, Barry MJ, Volandes AE: **Use of video to facilitate end-of-life discussions with patients with cancer: a randomized controlled trial.** *J Clin Oncol* 2010; 28: 305-311.
7. Kreuter MW, Holmes K, Alcaraz K: **Comparing narrative and informational videos to increase mammography in low-income African American women**. *Patient Educ Counsel* 2010; 81 S1: S6-S14 (2010)

8. Jibaja-Weiss ML, Volk RJ, Granchi TS: **Entertainment education for breast cancer surgery decisions: A randomized trial among patients with low health literacy** *Patient Educ Counsel* 2011; 84: 41-8.
9. Volk RJ, Jibaba-Weiss ML, Hawley ST, Kneuper S, Spann SJ, Miles BJ, Hyman DJ: **Entertainment education for prostate cancer screening: a randomized trial among primary care patients with low health literacy.** *Patient Educ Counsel* 2008; 73:482-489.
10. Fagerlin A, Wang C, Ubel PA: **Reducing the influence of anaecdotal reasoning on people's health care decisions: is a picture worth a thousand statistics?** *Med Decis Making* 2005; 25: 398-405.
11. Shaffer VA, Tomek S, Hulsey L: The effect of narrative Information in a publicly available patient decision aid for early-stage breast cancer. *Health Commun* 2013 [Epub ahead of print].
