# Supplementary material for: Do personal stories make patient decision aids more effective? A critical review of theory and evidence
Source: BMC Med Inform Decis Mak. 2013 Nov 29;13(Suppl 2):S9. doi: 10.1186/1472-6947-13-S2-S9 (PMC4044102; doi:10.1186/1472-6947-13-S2-S9)
Supplement: Additional file 3 — Table S3: Summary of Reviewed Studies’ Findings on Decision Processes and Outcomes [file 1472-6947-13-S2-S9-S3.pdf]

Table S3. Summary of Reviewed Studies' Findings on Decision Processes and Outcomes

Study	Summary of Results
Dillard et al. (2010) [1]	Hypothetical choice: colorectal cancer screening in adults.<br>Receipt of a narrative reduced perceived barriers to having the screening test, increased perceived risks of getting colorectal cancer, and increased interest in getting screened. No difference in knowledge.
Volandes et al. (2009) [2]	Hypothetical choice: end of life treatment for dementia in elderly adults.<br>Participants in the video aid group were more likely to choose comfort care (86% versus 64%), have higher knowledge scores and less likely to change their mind than those receiving verbal information alone.
Volandes et al. (2009b) [3]	Hypothetical choice: end of life treatment for dementia in elderly adults.<br>Receipt of the video narrative increased the proportion of elderly patients choosing comfort (conservative) care from 50-100%, and increased carers' awareness of the patient's treatment preferences from 33% to 100%.
Volandes et al. (2011) [4]	Hypothetical choice: end of life treatment for dementia in elderly adults.<br>Those viewing the video aid were more likely to choose comfort care (91% versus 72%).
Winterbottom et al. (2011) [5]	Hypothetical choice: peritoneal dialysis or haemodialysis in students.<br>Between 5-10% more participants chose the treatment option described by the 1st person (patient) narrative; there was no effect on treatment choice for the option described in the 3rd person (doctor) narrative.<br>Narratives delivered in video or written formats were equally effective.<br>The inclusion of a decision-attribute table did not ameliorate the effect of the 1st person narratives.
El-Jawahri et al. (2009) [6]	Hypothetical choice: end-of-life care in patients with malignant glioma.<br>Those viewing the video were more likely to prefer comfort care (91%) than those receiving verbal information (22%), and less likely to choose CPR (9% versus 41%). Knowledge was higher in the video group.
Kreuter et al. (2010) [7]	Women making mammogram decisions.<br>Women with less education were more likely to have a mammogram when viewing the narrative (65%) than information (32%) video. At 3 and 6 months, women's recall of information was higher in those who viewed the narrative (72/ 70%) than information (60/58%) video.<br>Women liked, and talked about, the narrative video more than women who saw the informational video, and had higher cancer beliefs and

	intentions to get a mammogram.
Jibaja-Weiss et al. (2011) [8]	Women making breast cancer treatment decisions.<br>Women viewing the decision aid were more likely to prefer radical mastectomy than women receiving the standard breast cancer information (60% vs. 40%). Pre-surgery, women receiving the decision aid had greater knowledge and reported making more informed decisions than those receiving the standard information.
Volk et al. (2008) [9]	Men engaging with education resources about prostate cancer screening at low and high literacy level healthcare sites.<br>Men in the low literacy site were more willing to seek screening information, were clearer about their screening values, and less conflicted by their screening decision if they received the narrative-based intervention; there were no differences in men from the high literacy sites.
Fagerlin et al. (2005) [10]	Hypothetical choice: angina treatment in adults.<br>20% chose bypass when one anecdote for both bypass or balloon angioplasty was presented, 41% when the number of anecdotes represented the statistical success of each option. When participants were presented with enhanced statistics, there was no anecdote effect.
Shaffer et al (in press 2013) [11]	Hypothetical choice: mastectomy or lumpectomy with radiation in women with no history of breast cancer.<br>52% of women chose mastectomy in both the control and narrative video groups. The reasons women gave for their treatment choice differed by video, those viewing the control video cited local recurrence for their choice, those viewing the narrative cited appearance. The narrative video was perceived as more emotional and trustworthy. Women who were more numerate found the control video to be more beneficial; those who were more computer literate found the narrative video to be more beneficial.

References

1. Dillard AJ, Fagerlin A, Zigmund-Fisher BJ, Ubel PA: **Narratives that address affective forecasting errors reduce perceived barriers to colorectal cancer screening.** *Soc Sci Med* 2010; 71: 45-52.
2. Volandes AE, Paasche-Orlow MK, Barry MJ, Gillick MR, Minaker KL, Chang Y, Cook EF, Abbo ED, El-Jawahri A, Mitchell SL: **Video decision support tool for advance care planning in dementia: randomised controlled trial.** *BMJ* 2009; 338: b2159.
3. Volandes AE, Mitchell SL, Gillick MR; Chang Y, Paasche-Orlow MK: **Using video images to improve the accuracy of surrogate decision making: a randomized controlled trial.** *JAm Med Dir Assoc* 2009b; 10; 575-580.
4. Volandes AE, Ferguson LA, Davis AD, Hull NC, Green MJ, Chang Y, Deep K, Paasche-Orlow MK: **Assessing End-of-Life Preferences for Advanced Dementia in Rural Patients Using an Educational Video: A Randomized Controlled Trial.** *J Palliat Med* February 2011, 14(2): 169-177.
5. Winterbottom AE, Bekker HL, Conner M, Mooney A: **Patient stories about their dialysis experience biases others' choices regardless of doctor's advice: an experimental study.** *Nephrol Dial Transplant* 2011; 27(1): 325-331.
6. El-Jawahri A, Podgurski LM, Eichler AF, Plotkin SR, Temel JS, Mitchell SL, Chang Y, Barry MJ, Volandes AE: **Use of video to facilitate end-of-life discussions with patients with cancer: a randomized controlled trial.** *J Clin Oncol*. 2010; 28: 305-311.
7. Kreuter MW, Holmes K, Alcaraz K: **Comparing narrative and informational videos to increase mammography in low-income African American women**. *Patient Educ Counsel* 2010; 81 S1: S6-S14 (2010)

8. Jibaja-Weiss ML, Volk RJ, Granchi TS: **Entertainment education for breast cancer surgery decisions: A randomized trial among patients with low health literacy** *Patient Educ Counsel* 2011; 84: 41-8.
9. Volk RJ, Jibaba-Weiss ML, Hawley ST, Kneuper S, Spann SJ, Miles BJ, Hyman DJ: **Entertainment education for prostate cancer screening: a randomized trial among primary care patients with low health literacy.** *Patient Educ Counsel* 2008; 73:482-489.
10. Fagerlin A, Wang C, Ubel PA: **Reducing the influence of anaecdotal reasoning on people's health care decisions: is a picture worth a thousand statistics?** *Med Decis Making*. 2005; 25: 398-405.
11. Shaffer VA, Tomek S, Hulsey L: The effect of narrative Information in a publicly available patient decision aid for early-stage breast cancer. *Health Commun* 2013 [Epub ahead of print].
